# Draft Genome Sequence of *Microbacterium profundi* Shh49^T^, an Actinobacterium Isolated from Deep-Sea Sediment of a Polymetallic Nodule Environment

**DOI:** 10.1128/genomeA.00642-15

**Published:** 2015-06-11

**Authors:** Yue-Hong Wu, Peng Zhou, Hong Cheng, Chun-Sheng Wang, Min Wu, Xue-Wei Xu

**Affiliations:** a Laboratory of Marine Ecosystem and Biogeochemistry, Second Institute of Oceanography, State Oceanic Administration, Hangzhou, China; b College of Life Sciences, Zhejiang University, Hangzhou, China

## Abstract

*Microbacterium profundi* strain Shh49^T^ was isolated from deep-sea sediment from a polymetallic nodule area located in the East Pacific Ocean. Strain Shh49^T^ contains genes related to the reduction/oxidation of metals. It has potential application in the bioremediation of heavy metal-contaminated environments.

## GENOME ANNOUNCEMENT

Ocean polymetallic nodules are widely distributed on the deep-sea floor and are a potential source of metals, such as Fe, Mn, Ni, Cu, and Co (1). Microbes participate in the formation of polymetallic nodules (2). *Microbacterium profundi* Shh49^T^ belongs to the class *Actinobacteria*. It was isolated from deep-sea sediment of a polymetallic nodule environment (8°22′38″N 145°23′56″W) at a depth 5,280 m (3). To understand the ecological function of strain Shh49^T^ and its potential role in the formation of nodules, the genome of strain Shh49^T^ was sequenced and analyzed.

Genomic DNA sequencing was performed using Solexa paired-end sequencing technology (HiSeq 2000 system; Illumina, Inc., USA) (4) by a whole-genome shotgun (WGS) strategy, with a 500-bp paired-end library (333 Mb available reads, 100-fold genome coverage) and a 2,000-bp paired-end library (140 Mb available reads, 42-fold genome coverage). All clean reads were assembled using SOAP*denovo* version 1.05 (5, 6). The quality of the sequencing read data was estimated by G+C content and sequencing depth correlation analysis. The tRNAs and rRNAs were identified using tRNAscan-SE (7), RNAmmer (8), and the Rfam database (9). The open reading frames (ORFs) and the functional annotation of translated ORFs were predicted and achieved using the RAST server online (10). The classification of some predicted genes and pathways was analyzed using the KEGG databases (11–13).

The draft genome sequence of strain Shh49^T^ revealed a genome size of 3,369,357 bp (scaffold length) and was assembled into 12 contigs. The G+C content was 66.54%. These scaffolds contain 3,269 coding sequences (CDSs), 44 tRNAs, and one copy of 16S-23S-5S rRNA gene operons. Among the protein-coding genes, 2,355 were assigned to putative functions, and the remaining were annotated as hypothetical proteins.

To study the ecological function of strain Shh49^T^ in the metal cycle, the reductase/oxidase relationship to metal reduction/oxidation, including Fe, Mn, Cu, and Hg, was analyzed. Four multicopper oxidases (MCOs), a family of enzymes known to be involved in Fe (14), Cu (15, 16), and Mn oxidation (17), were detected. Strain Shh49^T^ may have potential ability to oxidize iron from ferrous to ferric iron on the basis of the detection of two ferroxidases. Further experiments need to be performed to confirm its function. Strain Shh49^T^ encodes one mercuric ion reductase and one flavin adenine dinucleotide (FAD)-dependent NAD(P)-disulfide oxidoreductase, both of which participate in the reduction of Hg^2+^ to Hg. The genome of strain Shh49^T^ may further help us investigate the cycles of metals in deep-sea polymetallic nodules. Strain Shh49^T^ also has potential application in the bioremediation of heavy metal-contaminated environments.

### Nucleotide sequence accession number. 

The draft genome sequence of strain Shh49^T^ is available in GenBank under the accession no. JPSY00000000.
